# Micro-fragmented adipose tissue—An innovative therapeutic approach: A narrative review

**DOI:** 10.1097/MD.0000000000041724

**Published:** 2025-02-28

**Authors:** Hongjuan Fu, Congcong Wang

**Affiliations:** a Department of Anesthesiology, Yangguangronghe Hospital, Weifang, Shandong, China; b Department of Joint Surgery, Weifang People’s Hospital, Weifang, Shandong, China.

**Keywords:** adipose tissue, mesenchymal stem cells, micro-fragmented adipose, regeneration, therapeutic approach

## Abstract

Subcutaneous adipose tissue provides distinct advantages as a source of mesenchymal stem cells due to its accessibility and the ease of isolating stem cells. Human adipose stem cells, located in the stromal-vascular fraction, can be harvested using mechanical methods to produce microfragmented adipose tissue (MFAT). Local injections of MFAT have shown potential in promoting natural tissue regeneration. This review introduces the concept of MFAT, highlights its clinical applications, and explores its potential in regenerative medicine, offering insights into its role as an innovative therapeutic approach.

## 1. Introduction

Mesenchymal stem cells (MSCs) are vital in tissue repair, particularly for bone, cartilage, heart, vascular, and neural regeneration. Due to their extensive capacity for proliferation and differentiation, MSCs have become a key focus in cell-based tissue repair therapies. These cells can differentiate into various cell types, including adipocytes, chondrocytes, osteoblasts, and myoblasts.^[1–8]^ In addition to their differentiation potential, MSCs secrete a range of bioactive molecules that modulate immune responses and create an ideal environment for tissue regeneration.^[9–12]^

MSCs can be isolated from several tissues, such as dental pulp, fetal membranes, and adipose tissue (ASCs).^[13,14]^ Zuk et al were the first to identify a population of stem cells in human fat aspirates that exhibited similar potency and functionality to bone marrow-derived MSCs.^[3]^ Numerous studies have since confirmed that adipose tissue is an excellent MSC source due to its abundant cell yield and ease of access. One gram of adipose tissue can provide approximately 5000 stem cells—500 times more than bone marrow.^[15–19]^

This study does not require ethical approval because it is a systematic review of existing literature, and all data used were obtained from published studies that have already undergone ethical review by their respective institutions. No human or animal subjects were involved in the data collection or experimentation process.

## 2. Development of adipose tissue processing technology

**Figure 1.**
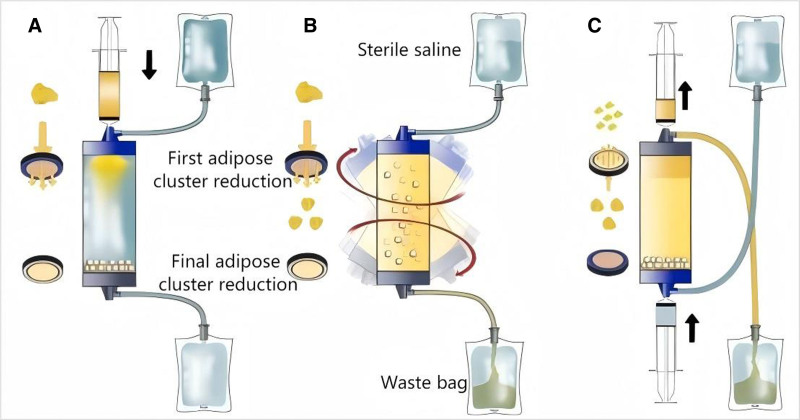
Schematic of the Lipogems device.

The predominant separation technique for adipose tissue involves enzymatic hydrolysis. Typically, enzymes such as collagenase, trypsin, or dispase are employed to digest adipose tissue. Although methods for isolating adipose-derived cells vary, the process generally begins with washing the adipose tissue, followed by enzymatic digestion, and isolation of cells by centrifugation from the oil and enzyme solution released by mature adipocytes.^[20]^ Due to the high costs and potential safety and efficacy concerns associated with enzymatic use,^[21,22]^ various research teams have focused on nonenzymatic separation techniques that employ shear, centrifugal, radiation, and pressure forces. This mechanical process supplants the use of enzymes for extracting individual cells or cell clusters from adipose tissue. The system used to process adipose tissue, initially developed by Dr Carlo Tremolada, named Lipogems, facilitates the harvesting, processing, and reinjection of adipose aspirates (Fig. 1).^[23]^ This system streamlines the surgical procedure. A minor skin incision is made under local anesthesia, and Klein solution is injected into the subcutaneous adipose tissue. The adipose tissue is then harvested and processed using the Lipogems device to obtain a product rich in MSCs. Throughout this process, the fat is subjected to only mild mechanical forces, which gradually reduce the adipose tissue clusters into micro-fragmented adipose tissue (ranging from spherical clusters of 1–3.5 mm in diameter to clusters of 0.2–0.8 mm), while eliminating oil and blood residues through a drain bag. The entire procedure is performed in a fully submerged system to minimize any invasive manipulation of the cell product. Approximately 100 to 130 mL of fat aspirate can be processed to yield about 60 to 100 mL of the final tissue product using the standard 225 mL Lipogems kit. The primary goal of the Lipogems system was to enhance the traditional Coleman fat grafting technique by providing transplantable fat clusters of reduced size for improved efficacy. Micro-fragmented adipose tissue obtained through other mechanical means is highly invasive to cells and produces substantial amounts of oil residue and cellular debris.

Lipogems technology produces micro-fragmented adipose tissue (MFAT) that is rich in mesenchymal stem cells by applying minimal mechanical force to liposuctions derived from human fat. The ASCs derived from MFAT meet the criteria for classification as mesenchymal stem cells but exhibit a more pronounced neural phenotypic profile.^[24]^ There is compelling evidence that these cells can differentiate into a diverse array of cell types, such as neuronal/glial-like cells, cardiomyocytes, endothelial cells, hepatocyte-like cells, various epithelial cell types, keratinocyte-like cells, and dental bud structures.^[25–34]^ Moreover, they can interact with immune system cells and have demonstrated immunomodulatory and anti-inflammatory effects.^[35–37]^ Lipogems products have shown enhanced transcription of angiogenic genes and significantly higher concentrations of exosomes compared to enzymatically dissociated cells, suggesting that these cells could be further optimized for their pluripotency.^[38–40]^ Exposure of Lipogems-derived ASCs to a radioelectric asymmetric conveyor has significantly enhanced the transcription of prostaglandins, GATA binding protein 4, NK2 Homeobox 5, vascular endothelial growth factor, hepatocyte growth factor, von Willebrand factor, neuroelement-1, and myogenic differentiation 1, indicating effects on the cardiac, vascular, neuronal, and skeletal muscle lineages.^[41,42]^ MFAT releases significantly more growth factors and cytokines involved in tissue repair and regeneration, particularly through angiogenesis, compared to the enzymatically derived stromal vascular fraction.^[43]^ MFAT contains a considerable amount of MSCs and possesses an impressive capacity to secrete molecules with anti-inflammatory properties, which remain active for weeks.^[44]^ The drain bag from the Lipogems system, in addition to containing oil and blood residues, also holds isolated cells that are readily expanded and exhibit typical features of ASCs. These cells can be loaded with paclitaxel to provide cell-mediated drug delivery.^[45]^ Local administration of MFAT loaded with paclitaxel offers a route for sustained-release chemotherapy, according to an animal study.^[46]^ In conclusion, the Lipogems technique enhances and optimizes the natural properties of adipose tissue. Instead of relying on enzymes, additives, or separation by centrifugation, the Lipogems system generates MFAT through gentle mechanical force, thus ensuring superior performance in treating joint injuries, plastic surgery applications, tissue trauma, gastrointestinal diseases, and other medical conditions.

## 3. Applications in various fields

### 3.1. Joint injury

The use of MFAT in joint injuries has gained significant attention due to its minimally invasive nature and potential to enhance natural tissue regeneration. Local injections of MFAT into damaged joints, particularly the knee and shoulder, have been shown to provide pain relief, improve joint function, and facilitate tissue repair. MFAT achieves these effects through multiple mechanisms: acting as a mechanical scaffold that supports the damaged cartilage, stimulating the activity of resident chondrocytes, and delivering MSCs capable of differentiating into cartilage cells to replace damaged tissue.^[47,48]^

One prominent case involved a 33-year-old skier who experienced persistent knee pain following anterior cruciate ligament reconstruction and micro-fracture surgery. After a platelet-rich plasma injection failed to provide long-term relief, the patient received an injection of MFAT. Within 10 days, significant pain relief was noted, and at a 30-month follow-up, the patient continued to report positive outcomes, highlighting the long-term benefits of MFAT in such cases.^[49]^ Similarly, studies have demonstrated that MFAT injections can effectively treat juvenile osteochondritis dissecans, with improvements in clinical scores such as International Knee Documentation Committee, Lysholm, and Tegner, as well as MRI findings.^[50–59]^

In clinical trials, MFAT has been shown to outperform other regenerative treatments, such as enzymatically derived stromal vascular fraction and expanded cells, in achieving optimal cartilage repair in osteoarthritis.^[60–64]^ For example, a large study involving 130 dogs with spontaneous osteoarthritis found that MFAT injections were safe and led to significant improvements in joint function.^[65]^ In human patients, Russo et al conducted a study on 30 individuals with knee osteoarthritis and reported a 20-point median improvement in subjective International Knee Documentation Committee and knee injury and osteoarthritis outcome scores, with most patients maintaining positive outcomes over a 3-year period.^[38]^ These findings have been supported by multicenter international studies showing that MFAT can improve pain, quality of life, and joint function, even in patients with advanced osteoarthritis.^[66–73]^

Despite its promising results, there have been rare cases of complications, such as MFAT migration. For example, one case reported that MFAT injected into the knee migrated into a Baker cyst, necessitating drainage for symptomatic relief.^[74]^ While these occurrences are infrequent, larger clinical trials are needed to fully assess long-term safety and efficacy, as well as to refine protocols for patient selection and treatment strategies.

### 3.2. Plastic, reconstructive, and aesthetic surgery

In the field of plastic and reconstructive surgery, MFAT has emerged as a promising technique for enhancing aesthetic outcomes and promoting tissue regeneration. Originally, adipose tissue was primarily used as a soft tissue filler due to its availability, low cost, and biocompatibility. However, with the discovery of the regenerative properties of adipose-derived MSCs, MFAT has expanded its role in aesthetic and reconstructive surgery. MFAT can be used both as a stand-alone therapy and in conjunction with traditional surgical techniques, such as facelifts, blepharoplasty, and scar revision, to improve healing, skin quality, and long-term outcomes.

In particular, MFAT has shown remarkable results in periorbital rejuvenation, where it is injected deep into the orbicularis oculi muscle to treat tear trough deformities and lower eyelid swelling. Patients typically report minimal pain, swelling, or bruising, and are highly satisfied with the aesthetic improvements.^[75]^ Beyond its role as a filler, MFAT contributes to long-term tissue remodeling, with improvements in skin texture, wrinkle reduction, and pigmentation correction observed over time.^[76–81]^

Moreover, MFAT is increasingly used in combination with orthognathic surgeries to accelerate postoperative recovery. By evenly filling the subcutaneous tissue, MFAT helps reduce swelling, minimize scarring, and enhance the overall aesthetic outcome. Compared to traditional fat grafting methods, MFAT is simpler to administer and results in more uniform distribution, making it an ideal complement to reconstructive procedures.^[82]^

### 3.3. Refractory soft tissue trauma

MFAT has shown great potential in treating chronic, nonhealing wounds and soft tissue injuries, which are often caused by diabetes, vascular disease, and autoimmune conditions. These wounds are notoriously difficult to manage due to poor vascularization and impaired healing processes. MFAT, with its anti-inflammatory and regenerative properties, offers a novel therapeutic option for these challenging cases.^[83–89]^

One of the most prevalent forms of refractory soft tissue injury is the diabetic foot ulcer, which leads to over a million amputations worldwide each year.^[90]^ Standard treatments, including surgical debridement and regional vascular reconstruction, often fail to heal these ulcers completely. However, clinical studies have demonstrated that MFAT can significantly enhance healing outcomes. In a randomized controlled trial involving 114 patients undergoing minor amputation due to diabetic foot ulcers, 80% of the MFAT-treated group experienced complete wound healing within 6 months, compared to only 46% in the control group.^[91]^ This result underscores the potential of MFAT to dramatically improve healing in patients at high risk of amputation.

Additional case reports highlight MFAT’s effectiveness in treating other types of chronic ulcers and nonhealing wounds. For example, a 56-year-old man with a 3-year history of a prepatellar ulcer saw remarkable improvement after MFAT treatment, with new skin forming within 4 weeks and complete resolution of pain.^[92]^ In another study, 17 patients with various types of chronic ulcers, including vascular ulcers, inflammatory ulcers, and bedsores, were treated with MFAT. Nearly all patients showed significant improvement, with complete healing in 7 patients and substantial wound reduction in 6 others.^[93]^

Animal studies further support MFAT’s role in enhancing angiogenesis and promoting tissue regeneration. For example, MFAT has been shown to upregulate the expression of vascular endothelial growth factor, Kinase Domain-containing Receptor, and hepatocyte growth factor, key factors involved in blood vessel formation and tissue repair, indicating its potential use in treating ischemic limbs and other vascular-related injuries.^[94–100]^

### 3.4. General surgery

Beyond its applications in orthopedics and wound care, MFAT is increasingly being explored in general surgery, particularly for the treatment of conditions such as fecal incontinence and perianal fistulas. Fecal incontinence, which can result from obstetric injury, trauma, or anorectal surgery, significantly impacts patients’ quality of life. MFAT offers a minimally invasive solution by promoting tissue regeneration and muscle repair.

In one study, 5 patients with fecal incontinence due to previous anorectal or pelvic surgery received MFAT injections around the external and internal anal sphincters, as well as the pudendal nerve. After 24 months of follow-up, all patients showed significant improvements in continence, with the Wexner Incontinence Score improving from a mean of 14.0 preoperatively to 3.4 postoperatively. Ultrasound examination also revealed evidence of muscle repair and regeneration.^[101]^

Perianal fistulas, particularly in patients with Crohn disease, present another challenging surgical condition. Current treatments, including biotherapy and surgical drainage, often have limited success, with cure rates below 60%.^[102]^ However, studies have shown that MFAT can be a promising alternative. In one pilot study involving 19 patients with complex anal fistulas, MFAT achieved a 73.7% cure rate, demonstrating its potential to address this difficult condition.^[103]^ Further research in this area is ongoing, with early results suggesting that MFAT could become a key tool in the management of perianal fistulas, particularly in patients with Crohn disease.^[104]^

## 4. Conclusions

Adipose tissue serves as an ideal source of MSCs due to its ease of access and the ability to harvest a sufficient quantity of cells with minimally invasive techniques. The Lipogems system enhances and optimizes the natural properties of adipose tissue by using gentle mechanical forces rather than enzymes, additives, or centrifugation, to produce MFAT. This approach preserves the biological integrity of the tissue and creates a regenerative environment conducive to tissue repair.

MFAT has demonstrated significant therapeutic potential across various medical fields, including joint injuries, plastic and reconstructive surgery, soft tissue trauma, and general surgery. In joint injuries, MFAT injections provide pain relief and functional improvements while promoting cartilage regeneration. In plastic surgery, MFAT accelerates healing and enhances aesthetic outcomes. It has also proven effective in treating chronic, nonhealing wounds, such as diabetic foot ulcers, by promoting tissue regeneration and reducing inflammation. Additionally, its application in general surgery has shown promising results in treating conditions like fecal incontinence and perianal fistulas.

Despite these promising findings, the clinical application of MFAT is still in the early stages. Current studies involve relatively small patient cohorts, and while the results are encouraging, larger, more robust clinical trials are needed to validate its long-term efficacy and safety. Further research should focus on optimizing MFAT extraction methods, understanding its mechanism of action in different tissues, and identifying the best strategies for its clinical application.

As the field of regenerative medicine continues to evolve, MFAT represents an exciting, minimally invasive option for tissue repair and regeneration. Its broad applicability across multiple medical specialties suggests that it could become a key tool in personalized medicine, offering patients improved outcomes with fewer complications.

## Author contributions

**Writing – original draft:** Hongjuan Fu, Congcong Wang.

**Writing – review & editing:** Congcong Wang.
